# The impact of comorbid chronic conditions on quality of life in type 2 diabetes patients

**DOI:** 10.1007/s11136-015-1061-0

**Published:** 2015-08-13

**Authors:** Marcel C. Adriaanse, Hanneke W. Drewes, Iris van der Heide, Jeroen N. Struijs, Caroline A. Baan

**Affiliations:** Department of Health Sciences and EMGO Institute for Health and Care Research, VU University Amsterdam, De Boelelaan 1085, 1081 HV Amsterdam, The Netherlands; Centre for Nutrition, Prevention and Health Services, National Institute for Public Health and the Environment, Bilthoven, The Netherlands

**Keywords:** Type 2 diabetes, Comorbidities, Quality of life, Primary care

## Abstract

**Objective:**

To study the prevalence, impact and dose–response relationship of comorbid chronic conditions on quality of life of type 2 diabetes patients.

**Research design and methods:**

Cross-sectional data of 1676 type 2 diabetes patients, aged 31–96 years, and treated in primary care, were analyzed. Quality of life (QoL) was measured using the mental component summary (MCS) and the physical component summary (PCS) scores of the Short Form-12. Diagnosis of type 2 diabetes was obtained from medical records and comorbidities from self-reports.

**Results:**

Only 361 (21.5 %) of the patients reported no comorbidities. Diabetes patients with comorbidities showed significantly lower mean difference in PCS [−8.5; 95 % confidence interval (CI) −9.8 to −7.3] and MCS scores (−1.9; 95 % CI −3.0 to −0.9), compared to diabetes patients without. Additional adjustments did not substantially change these associations. Both MCS and PCS scores decrease significantly with the number of comorbid conditions, yet most pronounced regarding physical QoL. Comorbidities that reduced physical QoL most significantly were retinopathy, heart diseases, atherosclerosis in abdomen or legs, lung diseases, incontinence, back, neck and shoulder disorder, osteoarthritis and chronic rheumatoid arthritis, using the backwards stepwise regression procedure.

**Conclusion:**

Comorbidities are highly prevalent among type 2 diabetes patients and have a negative impact on the patient’s QoL. A strong dose–response relationship between comorbidities and physical QoL was found. Reduced physical QoL is mainly determined by musculoskeletal and cardiovascular disorders.

## Introduction

Quality of life is an important patient reported outcome in the diabetes research field. Quality of life incorporates the patient’s perspective of his/her physical, mental and social well-being. The importance of achieving and maintaining a good quality of life is increasingly been recognized, and stated in diabetes guidelines, [1] and represents in general an important goal for health care on its own.

Poor quality of life is associated with adverse outcomes in people with type 2 diabetes, including poor response to therapy, disease progression, and cardiovascular mortality [2, 3]. Complications and comorbid conditions primarily determine the quality of life of diabetes patients [4]. A considerable proportion of diabetes patients develop disease related complications such as cardiovascular disease, nephropathy, retinopathy and neuropathy. However, non-diabetes related comorbid conditions are also common among people with diabetes [5]. Comorbidities can have profound effects on patient’s ability to manage their self-care and pose significant barriers to lifestyle changes and regimen adherence [6].

So far, the role of comorbidities on diabetes patient’s mental and physical quality of life is understudied. The number of studies that have reported both about the prevalence, impact and the dose–response relationship between comorbidity and quality of life in type 2 diabetes patients is limited. The interactions of diabetes and comorbid conditions are becoming increasingly important as the prevalence of individuals with diabetes and multiple chronic conditions increases [7, 8].

Therefore, our aim was to study the prevalence, impact and the dose–response relationship of comorbid chronic conditions on mental and physical quality of life in type 2 diabetes patients. A better understanding of the influence of comorbid conditions on quality of life in diabetes patients might also underscore the importance of preventing and treating comorbidity in individuals with diabetes. Ultimately, this may lead to improvements in the comprehensive management of diabetes patients and subsequently increase their quality of life.

## Research methods and design

### Study design and population

Data were derived from an observational study evaluating the effects of bundled payment on the healthcare delivery process and quality of diabetes care, provided by Dutch care groups. For the observational study, care groups were selected based on size, catchment area, geographical location and composition (e.g., rural vs urban), and their organizational structure. Care groups are groups of associated care providers, often exclusively general practitioners, who are responsible for coordinating and ensuring the delivery of services included in the disease management program [9]. Care groups can decide to either deliver the various diabetes care components themselves or subcontract other care providers. Detailed information about this payment reform, the related evaluation and the methods is published elsewhere [10, 11]. For the present study, we used data over a period from June 2009 to June 2010. The current study population consists of type 2 diabetes patients available from eight care groups in the Netherlands.

### Data collection

Data were collected from two data sources: patient medical records and patient survey questionnaires completed by patients enrolled in the disease management programmes of the care groups. As part of the observational study, a patient survey was administered to a random sample of 4377 diabetes patients clustered within a random sample of 78 general practices, which were subcontracted by eight care groups. A reminder was sent three weeks later to all who had not yet responded. A total of 1941 (response rate: 44 %) patient questionnaires were completed and returned. To link patients’ survey questionnaire data to medical record data, patient surveys were given pseudonymous identification numbers before they were distributed. Of the 1941 completed questionnaires 1714, (88 %) could be linked to patients medical records; 38 patients with type 1 diabetes cases were excluded. The final study sample consisted of 1676 type 2 diabetes patients.

### Quality of life

Quality of life was measured using Short Form-12 (SF-12). The SF-12 is a generic, reliable and validated instrument, containing 12 items derived from the Short Form-36 questionnaire [12]. Physical and mental quality of life was measured using the physical component summary (PCS) and mental component summary (MCS) scores of the SF-12, respectively. The PCS items include an assessment of the participant’s self-report on the level of limitations experienced in performing moderate activities, climbing stairs, accomplishing less because of physical health, the experience of bodily pain, and a rating of general health. The MCS items include questions on feeling calm and peaceful, downhearted and blue, accomplishing less, and doing activities less carefully than usual because of one’s mental health. The SF-12 is recommended for use in large surveys and has been used in diabetes populations before [13, 14]. Both the PCS and MCS score range from 0 (worse) to 100 (best). Dutch age- and sex-standardized population norms are available [15, 16]. In adults aged 60–69, the mean PCS score is 50.9 ± 8.3, and the mean MCS score is 51.3 ± 8.8 points [16]. A higher score in the respective summary scales represents a higher level of functioning. It is suggested that a minimum difference in three to five points is considered clinically important [17].

### Comorbid chronic conditions

The presence of comorbidities was determined through the patient questionnaire using a fixed list of 16 chronic conditions. This list was derived from the permanent survey on living condition (PSLC). The PSLC is yearly administered to a random sample of the Dutch population to gain insight into trends in health, medical consumption, and aspects of life style. The fixed list of conditions is developed under the auspices of Statistics Netherlands and has been applied regularly in health surveys in the Netherlands in the last decades [18]. Respondents were asked whether they had suffered from one of the following 16 conditions in the last 12 months: (1) retinopathy; (2) cerebral diseases (i.e., stroke, cerebral hemorrhage, cerebral infarct, or transient ischemic attack); (3) myocardial infarction; (4) severe heart disease (such as congestive heart failure or angina pectoris); (5) cancer/malignancies; (6) migraine or frequent severe headache; (7) high blood pressure; (8) atherosclerosis in abdomen or legs; (9) lung diseases (i.e., asthma, chronic bronchitis, lung emphysema or chronic obstructive pulmonary disease (COPD); (10) intestine disorder (severe and persistent for more than 3 months); (11) incontinence; (12) back disorder (severe/persistent); (13) osteoarthritis (hip, knee); (14) chronic rheumatoid arthritis; (15) neck, shoulder disorder (severe/persistent); and (16) elbow, wrist and hand disorder (severe/persistent).

### Other variables

Patient questionnaires and patient medical records were used to define the other variables. The self-administered patient questionnaire consisted of questions on education, ethnicity, smoking and physical activity. These variables were derived from standard questionnaires used in the Dutch National Health Survey. Education was categorized into: low (primary school, lower occupational education or less), medium (secondary level education) and high education (university, higher occupational or corresponding education). Smoking status was defined as currently smoking or not. Physical activity was assessed with two questions covering time spent on leisure time activities such as walking, bicycling, odd jobs, sports, and gardening and sport activities during the week. A summary score of both physical activity questions was calculated (range 0–7). Patient record data included general patient characteristics including sex, age, body mass index [calculated by dividing weight (kg) by height squared (m^2^)], HbA1c level, insulin use, diabetes duration (defined as number of years since diagnosis) and systolic blood pressure (SBP). The diagnosis of type 2 diabetes was obtained from medical records. Type 2 diabetes patients were diagnosed according to the World Health Organisation/International Diabetes Federation (WHO/IDF) 2006 criteria. The diagnosis was verified by the general practitioners.

### Missing values

Missing values were computed, for both patient registration data (i.e., HbA1c) and survey data (i.e., Quality of life) using multivariate imputation by chained equations (MICE) procedure in R [19]. Twenty imputation datasets were created. Analyses were performed with the multiple imputed datasets [20]. The necessary number of iterations for each missing value was 40 based on the Gibbs sampler [19]. Results of the analyses on the twenty imputed datasets were pooled by the MIANALYZE procedure in SAS.

### Statistical analysis

Descriptive data are presented for the total sample and type 2 diabetes patients with and without comorbidities. Differences in study sample characteristics for patients with and without comorbidities were examined using t tests for continue variables and Chi-square tests for dichotomous and categorical variables. Next, mean differences in mental (MCS) and physical (PCS) scores of quality of life by comorbid chronic condition in type 2 diabetes patients were calculated. These analyses were calculated and adjusted for age, sex, duration of diabetes, HbA1c, low-density lipoprotein cholesterol, systolic blood pressure, body mass index, insulin use, current smoking and physical activity. Thereafter, unadjusted and adjusted Beta coefficients for MCS and PCS scores of quality of life by number of comorbid chronic conditions [0 (reference category), 1, 2, 3, and 4 or more] among type 2 diabetes patients were calculated. Additionally, we performed analysis of variance (ANOVA) to test the differences in unadjusted and adjusted mean PCS and MCS scores by number of comorbid chronic condition. Finally, linear regression analyses were applied to determine the associations between comorbidities and PCS score in the group of diabetes patients with comorbidities only. The influence of comorbidities was analyzed for each comorbid chronic condition independently and in combination (backwards stepwise regression elimination procedure). The backwards elimination procedure has an advantage over forward selection and stepwise regression as it is possible for a set of variables to have considerable predictive capability even though any subset of them does not. Forward selection and stepwise regression would fail to identify them. Backwards elimination starts with all variables (i.e., comorbidities and demographics) in the model, so their joint predictive capability can be detected. For all statistics, we used two-sided hypotheses testing with an alpha level of 0.05. All analyses were performed using SAS (version 9.3, SAS Institute Inc, Cary, NC).

## Results

In total 1676 type 2 diabetes patients were included, aged 31–96 years (mean age 67.3 ± 11.1), of which 846 were male (50.5 %). Out of the total sample, 361 subjects (21.5 %) had no comorbid chronic conditions. The study sample characteristics and mean quality of life scores in diabetes patients with and without comorbidities are presented in Table 1. Diabetes patients with comorbidities were older, were more often females, had a higher body mass index, were more often smokers, were less physical active, had more often a Western ethnicity, and had higher systolic blood pressure, compared with diabetes patients without comorbidities, whereas HbA1c levels, low-density lipoprotein cholesterol levels and diabetes duration did not differ.

**Table 1:** Study sample characteristics and quality of life scores in type 2 diabetes patients with and without comorbidities

	Diabetes patients with comorbidities (*n* = 1315)	Diabetes patients without comorbidities (*n* = 361)	*P* value
Age (years)	67.8 ± 11.1	65.4 ± 10.9	<0.001
Male [*n* (%)]	615 (46.8)	230 (63.8)	0.028
Low education [*n* (%)]	635 (48.3)	128 (35.5)	<0.001
HbA1c (mmol/mol)	50.3 ± 10.3	50.5 ± 9.8	0.625
LDL (mmol/l)	2.4 ± 0.8	2.4 ± 0.8	0.678
Body mass index (kg/m^2^)	30.2 ± 5.5	28.6 ± 5.0	<0.001
Current smoking [*n* (%)]	188 (13.6)	304 (84.3)	<0.001
Physical activity (range 0–7)	3.5 ± 2.5	4.3 ± 2.6	<0.001
Western ethnicity [*n* (%)]	1266 (96.3)	333 (92.2)	<0.001
Systolic blood pressure (mm/hg)	138 ± 16.7	132.6 ± 9.8	<0.001
Diabetes duration (years)	7.4 ± 5.9	6.7 ± 5.1	0.166
Oral diabetes medication [*n* (%)]	959 (72.9)	268 (74.2)	<0.001
Insulin use [*n* (%)]	334 (25.4)	68 (18.8)	0.040
Quality of life (SF-12)			
PCS	42.6 ± 11.1	51.2 ± 7.9	<0.001
MCS	52.6 ± 9.2	54.5 ± 7.5	<0.001

Data are means (± SD) for continuous variables

*LDL* low-density lipoprotein cholesterol, *SF-12* the 12-item short form health survey, *PCS* physical component summary, *MCS* mental component summary

### Quality of life

Diabetes patients with comorbid chronic conditions showed significantly lower PCS (−8.5; 95 % confidence interval (CI) −9.8 to −7.3) and MCS scores (−1.9; 95 % CI 3.0 to −0.9), compared with patients without comorbidities. After adjustment, these associations attenuated slightly though remained statistically significant; PCS (−6.9; 95 % CI −8.0 to −5.7) and MCS scores (−1.6; 95 % CI −2.6 to 0.5; data not shown).

### QoL by comorbid chronic condition

High blood pressure, osteoarthritis and neck and shoulder disorders were the most common comorbid conditions among type 2 diabetes patients (Table 2). All comorbid conditions are associated with decreased physical QoL, with the exception of cancer/malignancies. Osteoarthritis, chronic rheumatoid arthritis and neck and shoulder disorder have the most negative impact on PSC with each mean difference scores of ≥−8.0 points. In contrast, 4 out of 16 chronic conditions showed no mean difference MCS scores. Migraine/severe headache and intestine disorders showed the most negative impact on MCS, with mean differences scores of −6.34 and −5.11, respectively. All other mean differences in MCS scores were not >−3.0, thereby not exceeding the clinical importance difference score of three to five points.

**Table 2:** Mean difference in mental (MCS) and physical (PCS) scores (PCS) by comorbid chronic condition in type 2 diabetes patients

Chronic condition	*N*	Mean difference*		Mean difference*	
		PCS	*P* value	MCS	*P* value
Retinopathy	162	−6.13	<0.001	−0.44	0.558
Cerebral diseases	56	−7.56	<0.001	−2.76	0.024
Myocardial infarction	41	−5.19	0.003	0.28	0.845
Heart diseases	124	−6.94	<0.001	−0.07	0.938
Cancer/malignancies	67	−1.24	0.370	−0.04	0.972
Migraine/severe headache	164	−5.36	<0.001	−6.34	<0.001
High blood pressure	645	−1.87	<0.001	−1.35	0.003
Atherosclerosis abdomen/legs	158	−8.00	<0.001	−1.67	0.028
Lung diseases	213	−7.13	<0.001	−1.81	0.006
Intestine disorders	132	−5.18	<0.001	−5.11	<0.001
Incontinence	309	−7.27	<0.001	−2.83	<0.001
Back disorder	301	−9.03	<0.001	−2.48	<0.001
Osteoarthritis	597	−8.56	<0.001	−1.59	<0.001
Chronic rheumatoid arthritis	232	−8.89	<0.001	−2.81	<0.001
Neck, shoulder disorder	324	−8.02	<0.001	−2.12	<0.001
Elbow, wrist, or hand disorder	266	−7.59	<0.001	−2.12	<0.001

* Mean difference reflects the mean MCS and PCS scores of type 2 diabetes patients with chronic conditions minus the mean MCS and PCS scores of type 2 diabetes patients without chronic conditions

*N* number, *PCS* physical component summary, *MCS* mental component summary

### Associations QoL by number of comorbid chronic conditions

Chronic conditions were highly prevalent in patients with diabetes, with 1.315 (78.5 %) diabetes patients being identified as having one or more comorbidity, and 387 (23.1 %) having four or more comorbidities (Table 3). As the number of comorbidities increased, both PCS and MCS unadjusted and adjusted scores significantly decreased, indicating lower physical and mental QoL. These effects were most pronounced for the PCS; unadjusted and adjusted PCS scores dropped 15.5 (95 % CI −16.8 to −14.0) and 13.3 (95 % CI −14.5 to −11.7) points, respectively, within patients with four or more comorbid conditions compared to patients without comorbid conditions. Additional Pearson’s correlations confirmed the negative association between the number of comorbidities increased, both PCS (*r* = −0.51) and MCS scores decreased (*r* = −0.17, both *P* < 0.0001), indicating lower physical and mental QoL.

**Table 3:** Unadjusted and adjusted Beta coefficients (95 % confidence interval) for mental (MCS) and physical (PCS) scores by number of comorbid chronic conditions among type 2 diabetes patients (*n* = 1676)

	Number of comorbid chronic conditions				
	1 *n* = 376 B (95 % CI)*	2 *n* = 317 B (95 % CI)*	3 *n* = 235 B (95 % CI)*	4 or more *n* = 387 B (95 % CI)*	*P* value
MCS unadjusted	−0.54 (−1.66 to 0.57)	−0.71 (−1.91 to 0.49)	−1.94 (−3.28 to −0.60)	−4.29 (−5.65 to −2.92)	<0.001
MCS adjusted**	−0.58 (−1.71 to 0.54)	−0.60 (−1.83 to 0.63)	−1.70 (−3.11 to −0.29)	−3.87 (−5.36 to −2.38)	<0.0001
PCS unadjusted	−2.68 (−3.91 to −1.45)	−5.95 (−7.34 to −4.56)	−10.27 (−11.72 to −8.82)	−15.39 (−16.76 to −14.03)	<0.001
PCS adjusted**	−2.36 (−3.56 to −1.17)	−4.98 (−6.35 to −3.61)	−8.91 (−10.37 to −7.46)	−13.06 (−14.47 to −11.65)	<0.0001

* Reference group is number of patients (*n*=361) without any comorbid chronic condition

** Adjusted for age, sex, duration of diabetes, HbA1c, low-density lipoprotein cholesterol, systolic blood pressure, body mass index, insulin use, current smoking and physical activity

### Multivariable association QoL by comorbid chronic conditions

Since the impact of comorbidities was most profound on the physical QoL of type 2 diabetes patients, we determined the association between comorbidities and PCS scores for each chronic condition independently and in combination (Table 4). The backwards stepwise multiple regression procedure showed that, retinopathy, heart diseases, atherosclerosis in abdomen/legs, lung diseases, incontinence, back, neck and shoulder disorder, osteoarthritis and chronic rheumatoid arthritis were negatively associated with PCS scores, with Beta’s ranging between −2.96 and −4.76 (all *P* < 0.0001). This model explained 29 % of the variance; adding demographics (age, sex, education) to the model resulted in explaining 31 % of the variance.

**Table 4:** Physical (PCS) scores and the relationship with multiple comorbid chronic conditions among type 2 diabetes patients

Chronic condition	Each comorbidity independently^a^			Multiple analysis^b^		
	*B*	(95 % CIs)	*P* value	*B*	(95 % CIs)	*P* value
Retinopathy	−3.65	(−5.19 to −2.11)	<0.001	−3.88	(−5.41 to −2.35)	<0.001
Cerebral diseases	−2.88	(−5.49 to −0.29)	0.029	–		
Myocardial infarction	−0.26	(−3.30 to 2.78)	0.867	–		
Heart diseases	−3.96	(−5.74 to −2.19)	<0.001	−4.25	(−5.97 to −2.52)	<0.001
Cancer/malignancies	0.43	(−1.89 to 2.74)	0.719	–		
Migraine/severe headache	−0.93	(−2.48 to 0.62)	0.238	–		
High blood pressure	−0.19	(−1.12 to 0.74)	0.695	–		
Atherosclerosis abdomen/legs	−4.42	(−5.99 to −2.84)	<0.001	−4.51	(−6.07 to −2.94)	<0.001
Lung diseases	−3.57	(−4.95 to −2.19)	<0.001	−3.66	(−5.03 to −2.28)	<0.001
Intestine disorders	−0.54	(−2.25 to 1.16)	0.536	–		
Incontinence	−3.08	(−4.29 to −1.87)	<0.001	−3.32	(−4.52 to −2.12)	<0.001
Back disorder	−4.55	(−5.82 to −3.29)	<0.001	−4.76	(−6.01 to −3.51)	<0.001
Osteoarthritis	−4.71	(−5.75 to −3.66)	<0.001	−4.74	(−5.78 to −3.70)	<0.001
Chronic rheumatoid arthritis	−2.74	(−4.20 to −1.28)	<0.001	−2.96	(−4.38 to −1.54)	<0.001
Neck, shoulder disorder	−2.77	(−4.06 to −1.49)	<0.001	−3.03	(−4.26 to −1.78)	<0.001
Elbow, wrist, hand disorder	−0.49	(−1.91 to 0.92)	0.495	–		

^a^Linear regression analysis was used to calculate unstandardized coefficients *B* and 95 % confidence interval (CIs) for each comorbid chronic condition independently

^b^A prediction model was calculated using a backwards stepwise regression procedure starting with all comorbid chronic conditions and then eliminating all variables which did not contribute (*P* > 0.1) to the model

We also applied the backwards regression procedure for the association between comorbidities and MCS scores, showing that only migraine/severe headache, intestine disorders and incontinence were negatively associated with MCS scores, with Beta’s of −4.07, −5.42 and −1.95 respectively (all *P* < 0.001), explaining 7 % of the variance (data not shown).

## Discussion

Our study showed that comorbidity is highly prevalent in type 2 diabetes patients and has a significant impact on both physical and mental quality of life, compared to those without. About one-fifth of the diabetes patients reported no comorbid disorder, and as many as 23 % had four or more comorbidities which is supported by other studies [21, 22]. Reduced physical QoL is mainly determined by musculoskeletal and cardiovascular disorders. We found that quality of life deteriorates significantly with increasing numbers of comorbid conditions.

Previous studies reported that stroke and ischemic heart disease, retinopathy, neuropathy and kidney disease have unfavorable effects on the QoL of diabetes patients [3, 23–25]. However, these studies were predominantly limited to one specific concordant disease [diseases that overlap with diabetes in their pathogenesis and management plans (e.g., cardiovascular diseases)]. We observed that type 2 diabetes patients with chronic conditions experienced reduced quality of life not only from cardiovascular disorders but also from musculoskeletal disorders. The magnitude of the reported QoL reductions varies between 3 and 5 points, and even larger mean differences were found for each comorbid condition separately, pointing at the clinical importance of the outcomes. The association between musculoskeletal disorders and diabetes has been described previously [26].

We also observed a substantial dose–response relationship between comorbidity and QoL, with physical QoL decreasing steeply with an increasing number of comorbidities. The magnitude of the dose–response relationship is striking: adjusted PCS scores dropped 13.3 points in patients with four or more comorbid conditions compared with patients without any comorbid condition, and this is 1.8 times the standard deviation. Very few studies have reported about the dose–response relationship between comorbidity and QoL in type 2 diabetes patients [27, 28]. Only Solli et al. found this relationship between diabetes complications and QoL, but one-third of the sample consisted of type 1 diabetes patients. The studies of both Solli et al. and Ose et al. differed regarding setting, methods, sample size and instruments used.

Recognizing the high prevalence of comorbidities and its strong association with poorer QoL is important for prioritization of care in adults with diabetes and comorbidity [8]. It has been suggested that patients with multiple chronic conditions are prone to receive incomplete, inefficient and ineffective care [6, 29]. Yet, the literature regarding the relationship between comorbidity and quality of life in diabetes is inconclusive. We previously published that there were no differences between diabetes patient with and without comorbidity in terms of provided care, achievement of clinical outcomes and perceived coordination and integration of care [30] though other studies suggested that the quality of care does differ between diabetes patients with and without comorbidities [22, 31].

This study, consisting of a large cohort of male and female type 2 diabetes primary care patients, gave a unique opportunity to investigate simultaneously the prevalence, impact and dose–response relationship between comorbid chronic conditions and QoL. We used a valid and reliable instrument for the primary outcome (QoL), including norm scores, and had detailed information on multiple comorbidities from both survey and administrative data. Yet, some study limitations need to be addressed. First, an important consideration in interpreting our results is that the current study employed cross-sectional data, so causal relationships between type 2 diabetes, comorbidity, and QoL cannot be established. Second, this study may not have fully captured the comorbid conditions that determine QoL. We used an existing and regularly used list that included sixteen chronic somatic conditions that are prevalent in at least 1 % of the Dutch population. Besides somatic comorbidity, there is a proportion of type 2 diabetes patients that suffer from psychiatric comorbidity. It is known that depression and type 2 diabetes often co-occur [32] and that depression negatively affects quality of life [33]. It is likely that including depression in the list of chronic conditions would have had detrimental effects on the mental QoL scores in our sample. On the other hand, assuming that depression is present in our diabetes sample one would expect to see MCS scores, in both diabetes patients with and without chronic conditions, that were more deviant from the norm MCS of people aged 60–69. However, we cannot exclude the fact that a proportion of patients could have been detected as positive for depression by using an appropriate screening tool. Third, we used self-reports to determine the chronic condition status. Self-report may be less reliable than medical records. The methods used to identify comorbid conditions could influences the prevalence figures [34]. The main reason for not using the medical records is that these co-morbidities were not well registered and registered ambiguously in the selected eight different care groups. For this reason these data were not requested. Therefore, we decided to use the self-report questionnaire. However, some studies suggested that self-report data predict QoL as well or even better than comorbidity data from medical records [35–37]. Fourth, there was no information available on the reasons for non-response. We were unable to perform a non-responder analysis since we do not have an informed consent of the non-responders to perform a linkage between their survey-id and the available registration data based on the larger observational study from which our study sample was derived. All respondents gave informed consent for linking the survey data to their medical records for the purpose of the study. Therefore, we were (only) able to assess whether the respondents in our survey sample were representative of the total study population. We compared both groups in terms of sex, diabetes duration and age, and no major differences emerged. Therefore, we expect no substantial bias for the primary outcomes and subsequent generalizability of the results. Yet, one cannot fully rule out the possibility that the non-responders could have had some impact on the results.

Finally, the present study was predominantly limited to a sample of patients with Western ethnicity, which may limit the generalizability. As yet, it is not clear whether the relationship between type 2 diabetes, comorbidity, and QoL is consistent across different ethnic populations. Interestingly, in adults with diabetes, ethnic minorities had better physical QoL than whites [38].

In conclusion, comorbidities among type 2 diabetes patients are highly prevalent, have a profound impact on the patient’s QoL, which deteriorates substantially with increasing numbers of comorbid conditions. Reduced physical QoL is mainly determined by musculoskeletal and cardiovascular disorders. The results stress de cumulative impact of comorbidity on the patient’s quality of life. It also shows that the illness burden experienced by diabetes patients is not only associated with diabetes itself and its concordant diseases, but in particular suffer from comorbidities that are unrelated with the pathogenesis and management plans of type 2 diabetes. Improved management of diabetes, including its allied comorbid chronic disorders, may ultimately lead to a better quality of life for the diabetes patient.
